# Clinical utility of serial analysis of circulating tumour cells for detection of minimal residual disease of metastatic nasopharyngeal carcinoma

**DOI:** 10.1038/s41416-020-0871-1

**Published:** 2020-05-06

**Authors:** Josephine Mun-Yee Ko, Vince Vardhanabhuti, Wai-Tong Ng, Ka-On Lam, Roger Kai-Cheong Ngan, Dora Lai-Wan Kwong, Victor Ho-fun Lee, Yun-Hoi Lui, Chun-Chung Yau, Chung-Kong Kwan, Wing-Sum Li, Stephen Yau, Chen Guo, Sheyne Sta Ana Choi, Lisa Chan Lei, Kenneth Chun-Ho Chan, Candy Chi-Shan Lam, Candy King-Chi Chan, Wei Dai, Pek-Lan Khong, Maria Li Lung

**Affiliations:** 1 Department of Clinical Oncology, University of Hong Kong, Hong Kong (Special Administrative Region), People’s Republic of China; 2 Department of Diagnostic Radiology, University of Hong Kong, Hong Kong (Special Administrative Region), People’s Republic of China; 3 Center for Nasopharyngeal Carcinoma Research, University of Hong Kong, Hong Kong (Special Administrative Region), People’s Republic of China; 4 Department of Clinical Oncology, Pamela Youde Nethersole Eastern Hospital, Hong Kong (Special Administrative Region), People’s Republic of China; 5 Department of Clinical Oncology, Queen Elizabeth Hospital, Hong Kong (Special Administrative Region), People’s Republic of China; 6 Department of Clinical Pathology, Pamela Youde Nethersole Eastern Hospital, Hong Kong (Special Administrative Region), People’s Republic of China; 7 Department of Oncology, Princess Margaret Hospital, Hong Kong (Special Administrative Region), People’s Republic of China

**Keywords:** Cancer genomics, Tumour biomarkers, Head and neck cancer

## Abstract

**Background:**

Nasopharyngeal carcinoma (NPC) is an important cancer in Hong Kong. We aim to utilise liquid biopsies for serial monitoring of disseminated NPC in patients to compare with PET-CT imaging in detection of minimal residual disease.

**Method:**

Prospective serial monitoring of liquid biopsies was performed for 21 metastatic patients. Circulating tumour cell (CTC) enrichment and characterisation was performed using a sized-based microfluidics CTC chip, enumerating by immunofluorescence staining, and using target-capture sequencing to determine blood mutation load. PET-CT scans were used to monitor NPC patients throughout their treatment according to EORTC guidelines.

**Results:**

The longitudinal molecular analysis of CTCs by enumeration or NGS mutational profiling findings provide supplementary information to the plasma EBV assay for disease progression for good responders. Strikingly, post-treatment CTC findings detected positive findings in 75% (6/8) of metastatic NPC patients showing complete response by imaging, thereby demonstrating more sensitive CTC detection of minimal residual disease. Positive baseline, post-treatment CTC, and longitudinal change of CTCs significantly associated with poorer progression-free survival by the Kaplan–Meier analysis.

**Conclusions:**

We show the potential usefulness of application of serial analysis in metastatic NPC of liquid biopsy CTCs, as a novel more sensitive biomarker for minimal residual disease, when compared with imaging.

## Background

Nasopharyngeal carcinoma (NPC) has its highest incidence in Southern China, including endemic regions like Hong Kong.^1^ Because of its innocuous symptoms, the tumour often progresses to advanced stages prior to diagnosis. This greatly reduces survival and the quality of life. NPC occurs with a peak age for diagnosis only in the upper 40s, when an individual is still in the prime of life. In Hong Kong young males less than 44 years of age, NPC is the top cancer in terms of incidence.^2^ Earlier diagnosis and better predictive markers for metastatic tumours are important in the quest to improve patient survival.^3^ However, the survival for NPC patients with recurrent or primary metastatic disease is dismal, with a median overall survival of 20 months.^4^

Most epithelial cancer patients die from distant metastasis. Circulating tumour cells (CTCs) may be considered as seeds of metastasis due to escape of the cancer cells into the bloodstream from both primary and metastatic tumours.^5^ Detection of rare CTCs in the blood provides an exciting opportunity for early sensitive detection of metastatic cancer cells. The clinical utility of CTCs to monitor treatment response and prognostication has been demonstrated for various metastatic cancers.^6–12^ The challenging task of enrichment and isolation of rare CTCs in the blood has greatly improved and heralds an exciting opportunity to utilise blood-based cancer diagnostics to monitor cancer progression and treatment response real-time.^13,14^ The technological advancement for use of microfluidics approaches and hydrodynamic centrifugal forces allows fast and efficient size-based isolation of viable and label-free CTCs after depletion of white blood cells.^15^ We embarked on pilot studies for CTC detection and enumeration for six cancer types including breast, lung, colorectal, gastric, liver and prostate cancer patient bloods. Our previous work established succinct workflows for CTC enumeration after enrichment.^16^ This CTC isolation system and platform provides a “liquid biopsy” for monitoring disease progression and offers the opportunity of non-invasive real-time monitoring of patients to determine the efficacy of treatment regimens.

NPC is a radiosensitive tumour. However, for patients with late diagnosis and lymph node metastasis, survival prospects remain poor. Distant metastasis increases mortality. Monitoring multiple lesions and distant metastatic solid tumours in advanced metastatic NPC patients by conventional invasive biopsies is not practically feasible and is inherently limited due to the intrinsic tumour molecular heterogeneity. Thus, we aimed to perform molecular analysis of CTCs to assess the clinical utility of non-invasive real-time monitoring of disease treatment in metastatic NPC. Numerous clinical studies utilise the plasma EBV DNA test for prognostication of advanced NPC.^17–19^ However, CTC studies of NPC are scanty.^11^ To date, there are no studies for serial longitudinal examination of patients during treatment to reveal the dynamics and characterisation of the molecular features of CTCs for NPC patients undergoing palliative chemotherapy. We assessed the usefulness of CTC enumeration before, during and post-treatment to complement the currently utilised plasma EBV and PET-CT imaging for assessment of treatment outcome. We determined whether the mutation load obtained by NGS analysis after CTC enrichment is useful as a biomarker for detection of minimal residual disease. This current study provides the first NPC serial CTC analysis study utilising the size separation and CD45 depletion approach for 21 metastatic NPC patients. We now show the potential clinical utility of CTC analysis and the more sensitive detection of minimal residual disease in patients with partial or complete responses at the end of treatment in this serial study of metastatic NPC patients accompanied by PET-CT imaging for assessment of tumour burden and plasma EBV DNA levels. Liquid biopsies are the source of CTCs and plasma EBV DNA, which provide complementary clinical utility for early detection of minimal residual disease in metastatic NPC patients to enable clinicians to implement more radical treatment regimens to improve patient treatment outcomes.

## Methods

### Subjects

From 2015 to 2018, 21 NPC patients with distant metastasis planned for palliative chemotherapy were prospectively recruited from three hospitals, Queen Mary (QM), Queen Elizabeth (QE) and Pamela Youde Nethersole Eastern (PYNE) Hospitals. Serial blood samples were taken at four different timepoints before, during and at the end of treatment, as shown in Fig. S1. During the palliative chemotherapy (CT) for six cycles, baseline blood was taken before treatment (CTC1), after two cycles of CT (CTC2), after three cycles of CT (CTC3), and at the end of CT (CTC4) to correlate with the interim and final PET-CT imaging, respectively. CTC2 was missed in patient 1 and CTC3 in patient 20. An additional follow-up timepoint CTC5 in patient 14 was collected for verification of molecular findings observed at post-treatment CTC4. A total of 83 samples (from 21 patients) were collected. As outlined in Fig. S1, three PET-CT scans were performed for the patients before, during and at the end of treatment. Guidelines from the European Organisation for Research and Treatment of Cancer (EORTC) were followed to define tumour response by PET-CT imaging. Complete metabolic response (CMR) is defined as complete resolution of FDG uptake in all lesions, partial metabolic response (PMR) has ≥25% reduction in the sum of SUVmax after more than one cycle of treatment, and progressive metabolic disease (PMD) is defined as having ≥25% increase in the sum of SUVmax or appearance of new FDG-avid lesions. Stable metabolic disease (SMD) is defined as being neither CMR, PMR, nor PMD. Table 1 and Fig. S1 show the demographics and clinical information of the recruited metastatic NPC patients. The median age of these patients was 57 with a range from 13 to 70; 81% (17/21) were males.

**Table 1:** Demographic and clinical information of 21 patients with disseminated NPC .

Patient No.	Age	Gender	Stage at initial diagnosis (TNM)	Distant metastasis site	PFS (Months)	Recurrence	Treatment outcome (PET3)	Survival
1	57	M	IVC (T3N3bM1)	Bone, LNs	4	Yes	SMD	
2	57	M	IVC (T3N3M1)	Bone	5	Yes	SMD	
3	70	M	III (T3N2M0)	Distant LNs	5	Yes	PMD	Dead
4	37	M	III (T3N2M0)	Bone, pleural, liver, LN	5	Yes	PMD	Dead
5	57	M	IVC (T1N3bM1)	Bone, liver, LNs	5	Yes	PMD	Dead
6	62	M	IVA (T4N2M0)	Liver, LN	5	Yes	PMD	
7	38	M	IVB (T2N3bM0)	Bone, LN	6	Yes	PMD	Dead
8	51	M	III (T3N1M0)	Bone, lung, LN	4	Yes	PMD	Dead
9	45	M	IVC (T3N3M1)	Bone, liver, LN	5	Yes	PMD	
10	59	F	IVC (T4N0M1)	Bone, liver	11 PD	Yes	PMR	
11	53	M	IVC (T4N3M1)	Bone, presacral nodule	12 PD	Yes	PMR	
12	69	M	IVA (T4N1M0)	Lung	25	No	PMR	Dead
13	52	M	III (T3N1M0)	Lung, LN	15 In remission	No	PMR	
14	38	M	III (T3N1M0)	Bone, liver, lung	10 PD	Yes	CMR	
15	64	F	IVC (T1N2M1)	Liver	24 PD	Yes	CMR	
16	59	M	IVC (T3N3bM1)	Liver, lung	8 PD	Yes	CMR	Dead
17	50	M	IVC (T3N2M1)	Liver	9 PD	Yes	CMR	
18	62	M	IVC (T3N3M1)	Lung	28 in remission	No	CMR	
19	41	F	III (T3N1M0)	Lung, LN	17 in remission	No	CMR	
20	63	F	III (T1N2M0)	Bone, lung	30 in remission	No	CMR	
21	13	F	IVC (T4N3M1)	Bone, liver	18 in remission	No	CMR	

### CTC isolation and enumeration

Collected blood samples were directly put into STRECK tubes and processed within 72 h. CTC enrichment utilised CTChip®FR1 biochip analysis performed on a ClearCell®FX1 System (Biolidics, Singapore) after lysis of red blood cells in 7.5 ml blood samples and centrifugation at 500 g at room temperature for 10 minutes, followed by resuspension of cell pellets.^15,16,20^ The output CTC enriched sample was loaded and fixed on a poly-l-lysine slide for subsequent enumeration of CTCs by immunofluorescence (IF) staining according to standard protocols. CTC isolation and enumeration were performed with pan-keratin (CK), which recognises keratins 4, 5, 6, 8, 10, 13 and 18. Cells were pre-hybridised with blocking solution and then hybridised to a cocktail of antibodies of pan-CK/EpCAM (Cell Signaling Technology, USA) and CD45 (BD Biosciences, USA) conjugated with Alexa-555 and APC secondary antibodies and stained with DAPI, as previously described. The cell images obtained from metastatic NPC sample analysis were captured on a fluorescence scanner, the Cytation 5 Cell Imaging Multi-Mode Reader (BioTeck, USA). The imaged CTCs were counted by imaging software using CellProfiler and CellProfiler Analyst, as previously described.^16^ CTCs were defined as nucleated cells positive for DAPI and CK/EpCAM IF staining, but negative for CD45 IF staining.

### Quantification of plasma EBV DNA by real-time PCR

For CTC samples collected for the serial analysis, the plasma EBV DNA was quantified by real-time PCR with the LightCycler 480 system (Roche), as previously described.^21^ The real-time PCR assay targeting the Bam HI-W region of the EBV genome was performed in duplicate and calibrated with an internal DNA standard using Namalwa, which is an EBV-positive cell line harbouring two copies of the EBV genome per cell.^22,23^ In blood sampling timepoint EBV1, a level of 1500 copies/ml was considered significantly elevated from the baseline blood of NPC patients for CTC analysis, as previously reported in an earlier NPC CTC study.^11^ In blood sampling timepoints EBV2–4, a cut-off of 60 copies/ml was used to define positive EBV status, as was consistently detected by all laboratories joining the NRG-HN001 NPC study from the National Cancer Institute.^24^

### Cell line DNA spike-in trial for CTC mutational profiling

DNA extracted from an oesophageal squamous cell carcinoma (ESCC) cell line (KYSE270) was utilised in this spike-in experiment. ESCC cell lines were cultured in RPMI, as previously described.^25^ KYSE270 cells and a Red Cross healthy donor’s blood DNA sample (RC2202) were used in the spike-in experiment. To explore the sensitivity of our NGS protocol for a scenario with low purity of CTC content, mixtures of different proportions of KYSE270 DNA to RC2202 DNA ranging from 100%, 10%, 5%, 2.5%, 1.25% and 0% were subjected to whole-genome amplification (WGA), according to the Qiagen REPLI-g Single Cell WGA protocol. An indexed library was constructed and hybridisation to the Roche Nimblegen NGS SeqCap EZ capture kit for target capture analysis and pair end 150 lllumina Hi-seq sequencing was performed, as previously described.^26^ This custom kit covers up to 7 Mb of 1366 genes, which are commonly involved in oncology, particularly in metastasis, and as druggable targets. The sequencing data were processed following the GATK recommendations.^27^ In brief, the sequencing reads were aligned to human genome (hg19) by Burrows-Wheeler Aligner (BWA) followed by the INDEL realignment and base quality recalibration. The quality of the sequencing data is summarised in Table S2. The somatic single nucleotide variants (SNVs) were identified by MuTect using a default setting.^26^ The germline variants including SNVs and small INDELs were detected using GATK HaplotypeCaller. The workflow for NGS mutational profiling was as previously published.^26^ For clinical CTC samples, 7.5 ml blood was utilised for CTC enrichment by the high purity run protocol according to the manufacturer instructions. The output was subjected to the NGS assay as described above for WGA, library preparation and bioinformatics analysis. The combined annotation dependent depletion (CADD) score was used to assess damaging effects of missense mutations.^28^ Only the damaging protein-altering mutations, missense mutations defined by the CADD score >15 were included in blood mutation load. Validation of mutations detected in CTC samples was performed by Sanger sequencing for 87.8% (35/41) of the tested mutations.

### Statistical analysis

The receiver operating characteristic (ROC) analysis was run to test the sensitivity and specificity of CTC1-4, EBV1-4 for progression-free survival (PFS) of mNPC. The CTC numbers and log10 transformed EBV DNA copy number/ml were used as continuous variables for the ROC analysis. Spearman’s rank-order correlation was used to assess the relationship between different CTC counts and log10 transformed EBV DNA amounts at different timepoints. Overall survival (OS) and PFS were measured from the date of baseline blood collection before palliative CT treatment to the last disease evaluation or until death/recurrence. The interim imaging assessment was scheduled midway during the treatment. Kaplan–Meier curves of probability of PFS were used to analyse survival data and examined the association of clinical parameters and biomarkers with PFS and OS. During the evaluation of the markers of change of EBV DNA, CTCs or combined both CTC4/EBV4, patients with positive status at both biomarkers, either one marker, or negative status at both, were classified as group 2, 1 and 0, respectively. Statistical analyses were performed by SPSS version 26 and a *p* < 0.05 was considered statistically significant.

## Results

### Isolation of label-free viable CTCs using spiral microfluidics enrichment

**Fig. 1:**
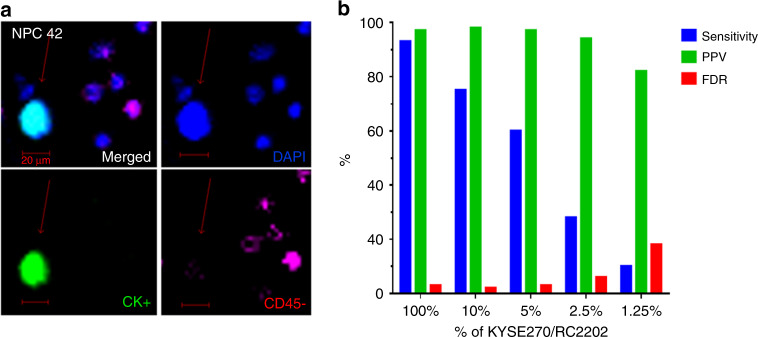
Circulating tumor cell detection by immunofluorescence staining and in spike-in experiments. **a** A representative immunofluorescence (IF) staining image of NPC CTCs after enrichment. **b** the positive predictive value (PPV), sensitivity, and the false discovery rate (FDR) of cell line spike-in experiments with KYSE270 and blood from a healthy individual (RC2202) at concentrations of 100%, 10%, 5%, 2.5% and 1.25% of the DNA content (All mutations detected by Mutect 1).

Previously, we established the CTC enrichment protocol based on cell-size separation, which is able to isolate viable and label-free CTCs in our laboratory using a spiral microfluidics CTC chip and the downstream workflow for CTC enumeration.^16^ Figure 1a shows the image of representative CTCs defined by DAPI+/CK+/CD45− staining from a NPC patient. The performance of this spiral system was tested by spike-in experiments with an ESCC cell line, (KYSE30) and a lung cancer cell line (H1975), with average cell diameter of 17 µm. Spike-in experiments with 100–300 CellTracker™ green-labelled KYSE30 cells into 7.5 ml blood from a healthy individual achieved a typical recovery rate ranging from 46% to 82% from eight independent default runs (average recovery rate = 60.6% ± 10.4%). Spike-in experiments were also performed with about 200 CellTracker™ green-labelled H1975 cells and achieved higher recovery rates ranging from 69.3% to 72.2% from three independent default runs (average recovery rate = 70.8% ± 1.5%).

### Establishment of workflow of SeqCap NGS mutation profiling for CTCs

The cell line spike-in quality control validation experiment demonstrated our established NGS protocol and pipeline was reliable for detecting the SNVs with a good sensitivity, positive predictive value (PPV > 97.0%), and low false discovery rate (FDR < 3.0%) from 10% down to 5% purity. The known mutation landscape of KYSE270 detected before and after WGA, and the mutations detected in various concentrations of KYSE270 in the background healthy donor RC2202 are shown in Supplementary Fig. S2. Somatic mutations were detected with high precision (>94%) with a minimum of 2.5% purity (Fig. 1b).

### Non-invasive serial monitoring of CTCs, EBV plasma DNA and PET-CT imaging in metastatic NPC patients

Among 21 metastatic patients, 11 (52.4%) were newly diagnosed patients suffering from synchronous distant metastasis at the time of diagnosis (i.e. IVC disease), while 10 patients developed de novo distant metastasis after initial radical treatment. The most common metastatic sites were bone, lung, liver and lymph nodes (LNs) (Table S1). After a median follow-up period of 21 months (range 7–37 months), the median PFS and OS were 9 and 21 months, respectively (Table 1).^29^ The results of serial analysis of EBV plasma, CTC counts, and imaging for all patients are summarised in Table 2. Clinical interim tumour assessment by imaging obtained by PET2, compared to PET1 taken at baseline, indicated that 2 patients had SMD, 14 had PMR and 5 patients had CMR. Tumour burden was evaluated by radiologic imaging at the end of treatment, as assessed by PET3. Nine (9/21, 42.9%) patients showed a poor response (2 with SMD and 7 with PMD) and 12 (12/21, 57.1%) showed good responses (4 with PMR and 8 with CMR). The EBV plasma assay, using the cut-off of 1500 copies/ml for elevated EBV status at baseline CTC blood collection,^11^ was positive for 17/21 (81%) cases before chemotherapy (CT) (EBV1). The EBV plasma assay using a lower stringent cut-off of 60 copies/ml for timepoints EBV2-4 was positive for 13/20 (65%) cases after two cycles of CT (EBV2), for 11/19 (57.9%) cases after three cycles of CT during treatment (EBV3) and for 11/20 (55%) patients at the end of treatment (EBV4). CTC enumeration, using the cut-off of ≥1 CTCs as positive, showed positive results for 11/20 (55%) patients at CTC1 before CT, 10/20 (50%) patients after two cycles of CT (CTC2), 11/18 (61%) patients at CTC3 after three cycles of CT, and 14/21 (67%) patients at the end of treatment (CTC4). The NGS blood mutation load analysis used a cut-off of >0.57 mutations per Mb based on our cell line spike-in experiments to detect positive minimal residual disease. We identified 13 NPC patients with positive blood mutation loads at the end of treatment by the NGS blood mutation load analysis.

**Table 2:** Serial analysis results of CTC counts, blood mutation load (BML), plasma EBV and imaging for 21 metastatic NPC patients.

Patients	# CTC samples	CTC	EBV (copies/ml)	Imaging	CTC count	# mutations	BML (#/Mb)^a^	PFS (months)
1	3	1	7838542	PET1	1	–	–	
		3	102813	PET2: PMR	4	–	–	
		4	172708	PET3: SMD	11	–	–	**Yes (4)**
		1	24958	PET1	NA	–	–	
2	4	2	386		0	–	–	
		3	452	PET2:PMR	NA	–	–	
		4	428	PET3:SMD	2	25	3.6	**Yes (5)**
3	4	1	173958	PET1	0	–	–	
		2	86094		0	–	–	
		3	41615	PET2: SMD	1	–	–	
		4	15896	PET3: PMD	2	66	9.4	**Yes (5)**
4	4	1	10895471	PET1	2	–	–	
		2	1191406		7	–	–	
		3	33203125	PET2: PMR	2	–	–	
		4	27791667	PET3: PMD	4	3	0.4	**Yes (5)**
5	4	1	394636	PET1	3	–	–	
		2	3268		18	–	–	
		3	3825	PET2: PMR	2	–	–	
		4	3922	PET3: PMD	2	113	16.1	**Yes (5)**
6	4	1	7109375	PET1	5	–	–	
		2	156458		2	–	–	
		3	385417	PET2: PMR	1	–	–	
		4	367083	PET3: PMD	2	25	3.6	**Yes (5)**
7	4	1	34740	PET1	1	–	–	
		2	41		0	–	–	
		3	102	PET2: PMR	0	–	–	
		4	2271	PET3: PMD	1	100	14.3	**Yes (6)**
8	4	1	504167	PET1	8	–	–	
		2	1103		5	–	–	
		3	654	PET2: PMR	4	–	–	
		4	2217	PET3: PMD	0	129	18.4	**Yes (4)**
		1	52792	PET1	3	–	–	
9	4	2	4879		4	–	–	
		3	812	PET2:PMR	8	–	–	
		4	1121	PET3:PMD	18	38	5.4	**Yes (5)**
10	4	1	42396	PET1	3	–	–	
		2	0		1	–	–	
		3	27	PET2: PMR	0	–	–	
		4	0	PET3: PMR	2	(a) 222	31.7	**Yes (11)**
						(b) 267	38.1	
11	4	1	75542	PET1	5	–	–	
		2	109		8	–	–	
		3	19	PET2: PMR	2	–	–	
		4	2329	PET3: PMR	4	46	6.6	**Yes (12)**
12	4	1	1204	PET1	0	–	–	
		2	0		0	–	–	
		3	0	PET2: PMR	0	–	–	
		4	0	PET3: PMR	0	21	3	Yes (25)
		1	94	PET1	0	–	–	
13	4	2	15		5	–	–	
		3	0	PET2:SMD	8	–	–	
		4	0	PET3:PMR	5	–	–	No (15)
14	5	1	341146	PET1	0	–	–	
		2	50		1	–	–	
		3	166	PET2: PMR	0	113	16.1	
		4	7	PET3: CMR	2	75	10.7	**Yes (10)**
		5	963		1	78	11.1	
						(b) 46	6.6	
15	4	1	123958	PET1	0	–	–	
		2	86		0	–	–	
		3	0	PET2: PMR	1	–	–	
		4	0	PET3: CMR	1	3	0.4	**Yes (24)**
16	4	1	399583	PET1	0	–	–	
		2	288		0	–	–	
		3	648	PET2: PMR	–	–	–	
		4	119	PET3: CMR	4	–	–	**Yes (8)**
17	4	1	20427	PET1	2	–	–	
		2	0		0	–	–	
		3	0	PET2: CMR	0	–	–	
		4	53	PET3: CMR	0	40	5.7	**Yes (9)**
18	4	1	40818	PET1	2	–	–	
		2	93		0	–	–	
		3	11	PET2: CMR	0	–	–	
		4	0	PET3: CMR	0	2	0.3	No (28)
19	4	1	899	PET1	0	–	–	
		2	0		4	–	–	
		3	0	PET2: CMR	0	–	–	
		4	0	PET3: CMR	0	160	22.9	No (17)
		1	165	PET1	0	–	–	
20	3	2	666		0	–	–	
		3	–	PET2:CMR	–	–	–	No (30)
		4	35	PET3:CMR	0	–	–	
		1	230417	PET1	0	–	–	
21	4	2	217		0	–	–	
		3	0	PET2:CMR	1	–	–	
		4	4	PET3:CMR	2	–	–	No (18)

PET1, PET2 and PET3 refer to the baseline, middle (before 3rd cycle) and end of treatment, respectively. The high purity runs for CTC enrichment of patients 1, 13, 16, 20 and 21, were not applicable for NGS analysis. Sufficient blood samples were available for duplicate BML analysis for patients 10 and 14.

^a^BML > 0.57 is defined as positive (four mutations were set as the cut-off in 7 Mb capture regions in the cell line spike-in experiment).

Patient died were shown as bold values in the table.

### Concordant imaging and liquid biopsy analysis for metastatic NPC patients with poor treatment response

**Fig. 2:**
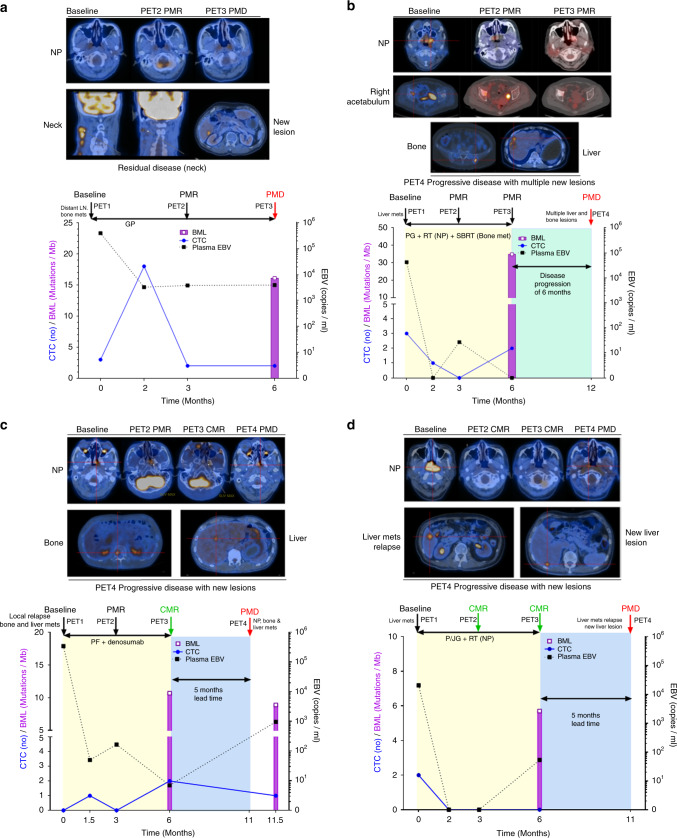
Representative longitudinal liquid biopsy monitoring and imaging for NPC patients having different treatment outcomes. **a** PET-CT imaging of NPC patient 5 showed partial metabolic response (PMR) in the middle and progressive metabolic disease (PMD) at the end of treatment. Serial liquid biopsy monitoring by plasma EBV1-4, CTC1-4 enumeration and CTC4 blood mutation load (16.1 mutations/Mb) all remained positive during treatment. **b** CTC serial monitoring findings of NPC patient 10 showed concordant results of disease burden compared to the PMR result of radiologic imaging in contrast to the negative mid- and post-treatment plasma EBV results. CTC2 and CTC4 enumeration data remained positive during and at the end of treatment and the CTC NGS assay detected 31.7 mutations/Mb in CTC4. **c** Radiologic imaging for NPC patient 14 showed complete metabolic response (CMR), but the CTC4 enumeration detected positive CTC counts and NGS analysis, with a high blood mutation load (6.6 mutations/Mb), providing a lead time of 5 months prior to imaging showing minimal residual disease; in contrast the EBV4 results only showed 7 EBV copies/ml. **d** The minimal residual disease of NPC patient 17, with CMR and ambiguous EBV findings, indicated by both the EBV4 plasma assay (53 copies/ml) and blood mutation load of CTC4 (5.7 mutations/Mb).

Liquid biopsy longitudinal real-time disease monitoring with the EBV assay, the CTC enumeration or NGS analysis was able to track the tumour burden in the nine patients (1–9) with poor treatment outcome (Table 2). Patient 5 was a stage IVC patient diagnosed with synchronous multiple organ metastasis treated with palliative chemotherapy. Concordant results of imaging and the three liquid biopsy molecular findings for this patient with a poor response are shown in Fig. 2a.

### CTC analysis offers an alternative means for tracking tumour burden for metastatic NPC patients with partial response

PET-CT imaging indicated four NPC patients had a partial response at the end of treatment; patient 11 was concordantly positive for tumour burden with both EBV and CTC findings, while patients 10, 12 and 13, in contrast, were three PMR patients with a negative EBV status during and at the end of treatment (Fig. 2b and Table 2). These data suggest that CTC analysis may offer an alternative liquid biopsy option to detect the residual disease for NPC patients with negative or ambiguous EBV findings. Patient 10 was diagnosed with stage IVC NPC and the disease progressed with detection of bone and liver metastases. The serial CTC molecular findings were consistently positive and showed concordance with radiologic imaging and clinical disease progression was detected six months after the end of treatment despite a discordant negative tumour burden indicated by plasma EBV2-4 findings (Fig. 2b). Two independent runs for CTC4 showed reproducible high blood mutation loads of 31.7 and 38.1 mutations/Mb (Table 2). Another PMR metastatic NPC patient 13 was diagnosed with stage III NPC with relapse in the lung and retropharyngeal lymph node metastases. Uncommonly, the EBV copies of patient 13 remained below 100 copies/ml at baseline and the EBV2-4 plasma findings showed discordant results with imaging. However, the serial CTC monitoring results changed from a negative baseline CTC counts to positive at CTC2 (5 counts) and CTC3 (8 counts) during treatment and at CTC4 (5 counts) at the end of treatment, indicating CTC enumeration may be useful for detection of disease burden in this patient (Table 2).

### Complementary role of plasma EBV DNA and CTC analysis for sensitive detection of early disease relapse for metastatic NPC patients compared with imaging

Imaging assessment at the end of CT detected complete response for eight NPC patients (14–21), but of these, four patients relapsed after 8–24 months (patients 14–17) (Table 2). The data suggest that the liquid biopsy, including EBV plasma findings and CTC molecular analysis, provide a more sensitive means of detection of minimal residual disease for early disease relapse compared to imaging. Three of these eight CR patients relapsed within one year. Patient 16 relapsed with the shortest PFS of 8 months. At the end of treatment of patient 16, both liquid biopsy findings from the EBV4 (119 copies/ml) and CTC4 status (four counts) were positive and indicated a minimal residual disease three months earlier than the clinically detectable disease progression. The early relapse after 9 and 10 months of patients 14 and 17 were complementarily shown by the plasma EBV and CTC analysis of minimal residual disease. The dynamics of molecular findings of liquid biopsy and imaging for patient 14 are summarised in Fig. 2c and patient 17 in Fig. 2d. Patient 14 was diagnosed with stage III NPC with disease progression after 2 years with local relapse and distant liver, lung, and bone metastases. Clinical radiologic evidence of bone and liver metastasis was detected after 5 months. A CTC5 follow-up timepoint was collected for liquid biopsy analysis to confirm the tumour burden and showed positive EBV (963 copies/ml) and blood mutation load (6.6–11.1 mutations/Mb) results (Table 2). Patient 17 was diagnosed with stage IVC NPC with liver metastasis. Post-treatment liquid biopsy analysis demonstrated 5 months lead time for more sensitive and early detection of minimal residual disease at the end of treatment compared to imaging (Fig. 2d). Patient 15 relapsed after two years with a positive post-treatment CTC4 detection (Table 2).

The evidence from serial monitoring suggested that positive findings of CTCs at the end of CT treatment for patients with the good short-term treatment outcomes indicate the potential clinical utility of CTC analysis being a more sensitive biomarker compared to imaging. Two NPC patients (19 and 21) with post-treatment radiologic imaging showed CMR and negative evidence of NPC disease from plasma EBV DNA, but the tumour burden was supported by the positive CTC analysis, remained progression-free for 1.5 years. The post-treatment blood mutation load of patient 19 was 22.9 mutations/Mb. The post-treatment CTC count of patient 21 was positive. These two patients require a longer follow-up to verify the post-treatment positive CTC mutation load suggestive of minimal residual disease.

Two NPC patients (18 and 20) had a CMR with the longest disease-free survival (28 and 30 months) and negative testing results from both plasma EBV DNA and CTC liquid biopsies for disease burden (indicated by three molecular findings; negative EBV4, CTC4 and CTC4 blood mutation load).

### Sensitivity and specificity of CTCs and EBV DNA load in mNPC

**Fig. 3:**
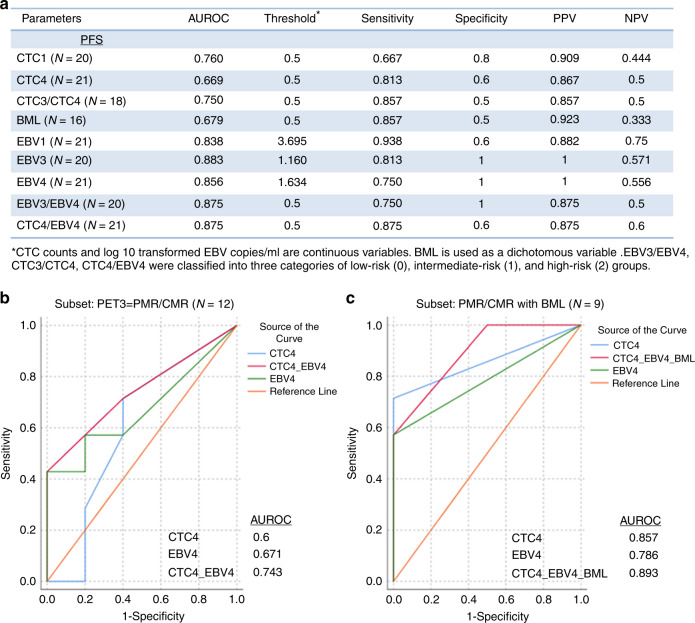
Sensitivity and specificity of longitudinal CTC and EBV DNA assays in mNPC. **a** Summary of AUROC, sensitivity, specificity, positive and negative predictive values (PPV and NPV) of CTCs and EBV DNA load for PFS in mNPC. **b** ROC analysis to test the specificity and sensitivity of CTC4, EBV4, combined CTC4/EBV4 and CTC4/EBV4/BML for PFS in mNPC patients with favourable response.

The sensitivity, specificity, positive predictive value (PPV) and negative predictive value (NPV) of CTCs and EBV DNA load in mNPC with PFS are summarised in Fig. 3a. Baseline EBV1, mid-EBV3, post-treatment EBV4, change of EBV3/EBV4 and combined CTC4/EBV4 are good PFS prognostic markers with the AUROC between 0.8 and 0.9. Baseline CTC1 and the change of CTC3/CTC4 are fair PFS prognostic markers with the AUROC between 0.7 and 0.8. For the 12 mNPC having good response (PMR or CMR) by imaging assessment taken at the end of treatment, combination of CTC4 and EBV4 (both negative = 0, either one positive = 1, both positive = 2), the AUROC is improved (0.743) compared with CTC4 or EBV4 alone (Fig. 3b). In a subset of nine mNPC patients, the AUROC of CTC4/EBV4/BML considering additional BML information (any two of CTC4/EBV4/BML are positive = 2, any one positive = 1, all negative = 0) is also improved (0.893) compared to CTC4 and EBV4 alone (Fig. 3c).

### Association of CTC counts with PFS

**Fig. 4:**
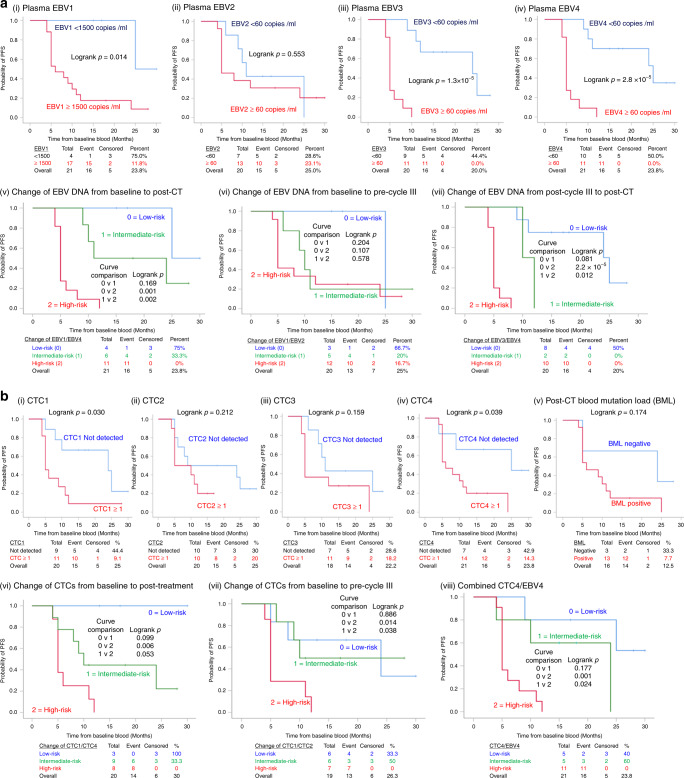
Kaplan–Meier analysis of progression-free survival (PFS) with EBV status, CTC enumeration and blood mutation load (BML) in serial and longitudinal analyses. Kaplan–Meier survival analysis of association of PFS with **a** serial EBV status (i–iv) and longitudinal change of EBV status (v–vii) at baseline, during and end of CT, and **b** serial CTC count status (i–iv), post-CT blood mutation load (BML) (v), longitudinal change of CTC status (vi–vii) at baseline, during and end of CT, and combined CTC4/EBV4 (viii).

Based on the log rank test, plasma EBV status at baseline, during and at the end of treatment were associated with PFS significantly for EBV1, *p* = 0.014, EBV3, *p* = 1.3 × 10^−5^, and EBV4, *p* = 2.8 × 10^−5^) (Fig. 4a (i–iv)). Similar associations were observed at baseline CTC1 and post-treatment CTC4 by Kaplan–Meier PFS analysis (log rank test, CTC1, *p* = 0.030 and CTC4, *p* = 0.039), whereas CTC2, CTC3, and blood mutation load at CTC4 did not reach statistical significance (*p* = 0.21, 0.16, 0.17, respectively) (Fig. 4b (i–v)). CTC2 taken at pre-cycle III CT treatment did not correlate with the interim imaging.

### Longitudinal changes in CTCs and EBV DNA along CT treatment for mNPC is predictive for disease relapse

A Spearman’s rank-order correlation was run to assess the relationship between baseline, during and post-treatment CTC counts in twenty-one mNPC cases. There were statistically significant positive correlations between CTC1/CTC2 (*ρ* = 0.564), CTC2/CTC3 (*ρ* = 0.663), CTC3/CTC4 (*ρ* = 0.692), EBV1/EBV2 (*ρ* = 0.653), EBV2/EBV3 (ρ = 0.850), EBV3/EBV4 (*ρ* = 0.862), EBV1/EBV4 (*ρ* = 0.661). The correlation between CTC1/CTC4 was low (*ρ* = 0.117). The high-risk group includes patients with detectable CTCs at both timepoints, the low-risk group includes patients with undetectable CTCs at both timepoints, and others were classified as intermediate-risk group. The same procedures were performed with EBV DNA changes and results are shown in Fig. 4a (v–vii). The median PFS of the low-risk and intermediate-risk groups stratified by EBV1/EBV4 change (Fig. 4a (v)) and EBV3/EBV4 change (Fig. 4a (vii)) were significantly longer than that of the high-risk group. The median PFS of the low-risk and intermediate-risk groups stratified by CTC1/CTC4 change (Fig. 4 Fig. 4 (vi)), CTC1/CTC2 change (Fig. 4b (vii)), and CTC4/EBV4 (Fig. 4b (viii)) were significantly longer than that of the high-risk group, although only a trend with marginal significance was observed for intermediate-risk vs high-risk groups for PFS and CTC1/CTC4. The change of CTC counts from baseline to pre-cycle III (Fig. 4b (vii)) can better predict the disease progression between intermediate-risk and high-risk group for PFS compared to that of EBV1/EBV2 DNA change (Fig. 4a (vi)).

## Discussion

NPC tumours are highly heterogeneous and heavily infiltrated with lymphocytes. Limitations of procuring adequate NPC tissues due to size limitations and tissue heterogeneity, as well as performing costly, invasive repeated tumour biopsies, hamper the understanding of NPC molecular pathogenesis of metastatic disease and the underlying mechanism of disease relapse. Non-invasive liquid biopsies utilise easily accessible blood samples to provide an alternative opportunity for real-time monitoring of disease progression and treatment response. Isolation of CTCs from the blood is a technical bottleneck for its clinical application.^14^ We recently established a platform for isolation of label-free CTCs based on size separation. We now demonstrate the feasibility of utilising liquid biopsies for CTC enumeration and NGS analysis to obtain the blood mutation load and comparative plasma EBV assay for monitoring the efficacy of chemotherapeutic treatment in NPC patients having distant metastasis. The EBV plasma status collected at the middle and end of treatment was a good independent predictive biomarker for treatment response representative of the tumour bulk and significantly associated with PFS in metastatic NPC patients.^17,30^ We validated previous observations that plasma EBV DNA findings provide good prognostic information of PFS and treatment outcomes of distant metastasis in NPC. The liquid biopsy analysis by CTC enumeration or blood mutation loads detected minimal residual disease in 75% (6/8) NPC patients (patients 14–17, 19 and 21) at the end of palliative CT, showing CMR by imaging. Post-treatment plasma EBV findings detected minimal residual disease in 62.5% (5/8) NPC patients with marginally positive readings of 7, 119, 53, 35 and 4 copies per ml (patients 14, 16, 17, 20 and 21). Our studies show that liquid biopsy CTC molecular findings provide a more sensitive means for earlier detection of residual tumour burdens compared to imaging. The current study for the first time suggests the potential clinical application of examining the blood mutation load of CTCs and serial CTC enumeration, which can complement commonly utilised plasma EBV DNA detection used at the completion of chemotherapy for patients with metastatic NPC. This is particularly relevant for the management of those patients found to have negative or only low-positive EBV DNA levels just marginally raised above the cut-off level. Our findings show the post-treatment CTC test is a potentially more sensitive tool to detect minimal residual disease in those good responders with a negative EBV test (Fig. 3b, c). In this study, several patients with CMR assessed by PET-CT imaging and negative or marginally positive EBV DNA levels after treatment, were positive for blood mutation load five months prior to showing PMD at the end of CT by PET-CT imaging and a clearly positive EBV DNA test result. A larger cohort study is needed to evaluate CTC tests as a secondary tool in risk stratification, when primary plasma EBV tests are negative. Providing clinicians with minimal residual disease results will allow timely clinical decisions to be made for more radical treatment strategies to facilitate optimal personalised medicine with health and economic benefits for patients. Predictive biomarkers to monitor treatment efficacy, apart from the conventional radiological imaging studies and EBV DNA assay, will provide the oncologist/clinician additional information to assist their decisions on offering additional local treatment after palliative chemotherapy for patients with stage IVC cancer.

Recent CTC studies now show that CTC enumeration in breast, colon, and prostate cancer patients are sensitive indicators of disease status and are even more sensitive and quantitative than conventional MRI and PET-CT scans or serological detection of cancer biomarkers.^31–33^ To date it is uncertain how accurate the use of imaging, including using the EORTC criteria, is for response assessment of NPC. For metastatic NPC patients, post-treatment CTC enumeration and blood mutation load analysis allowed sensitive detection of minimal residual disease for 75% of the good responders. Post-treatment CTC analysis may be potentially a useful tool for tracking tumour burden for low plasma EBV DNA NPC patients such as patients 12, 13, 19 and 20 in this study. Two NPC patients, 18 and 20, with negative findings from both the post-treatment plasma EBV DNA and the CTC analysis remained progression-free for more than two years. This observation suggests the potential role of liquid biopsy in stratification of metastatic patients with a favourable prognosis. However, we cannot rule out the possibility that CTC detection may not be specific for the cancer in patients 19, and 21. In this initial longitudinal serial monitoring of metastatic NPC patents, we established a workflow of blood-based diagnostic detection of both CTCs and plasma EBV DNA from stage IVC NPC patients to provide clinicians with additional information for prediction of treatment efficacy. We aim to identify the gene signature that serves as an early predictive biomarker for treatment decisions. Our preliminary data suggest that genes related to chromatin remodelling occur more frequently in the CTC samples from the group of patients with early disease relapse (PMD, *N* = 6) within 6 months compared to those with complete response (CMR, *N* = 5). Future studies with larger patient cohorts including advanced locally recurrent NPC patients at stages III, IVA and IVB are ongoing and will be useful to determine the clinical utility of blood-based real-time monitoring of tumour progression for personalised medical care of advanced NPC patients.

Mechanistic CTC studies from glioblastoma, breast and liver cancers, suggested aggressive subpopulations of CTCs detected in the blood circulation have invasive mesenchymal characteristics.^34–36^ Although nine (9/21, 42.9%) metastatic NPC patients had zero CTC counts before treatment, this finding may be attributed to the lack of detection by immunocytochemistry of CTCs not expressing epithelial markers for CTCs likely undergoing EMT^35^ or for CTCs of smaller size, which may not be captured. This also partially explains the discrepancy between the post-treatment CTC counts and blood mutation loads for some patients. One possible reason for finding two positive post-treatment CTC enumerations with negative blood mutation load may be due to fragility of CTCs, which may compromise DNA integrity and hinder their capture for NGS analysis. Further efforts for the search of tumour-specific markers are necessary for improvement of the CTC enumeration sensitivity and specificity.

The current data suggest that CTC analysis can provide complementary information for detection of minimal residual disease, when the EBV results are ambiguous and PET imaging indicate PMR or CMR at the end of the palliative CT for NPC patients with disseminated disease. Based on the current dataset, we find that CTC analysis at the end of treatment is useful for sensitive minimal residual disease detection and warrants further validation in a larger sample cohort with longer follow-up times to determine its clinical usefulness. The current study is limited by the small sample size and the follow-up time. We report data as a pilot study for generation of the hypothesis for further validation with larger sample size on the potential clinical utility of CTC analysis for good responders of metastatic NPC. The current study establishes a genomic profiling approach for CTCs by NGS target capture sequencing for identification of the molecular signature for metastatic progression and tracking molecular events occurring during the tumour evolution under treatment pressure. Genomic mutational analysis of CTCs will provide strategic information on key genetic alterations hallmarking metastatic tumours. Serial monitoring of patients during treatment will provide useful information on tumour evolution and the development of drug resistance. Incorporation of the non-invasive liquid biopsy CTC assay may obviate the need of invasive tissue biopsy and radiological imaging for monitoring and predicting cancer progression and for guiding treatment choices. Our long-term goal is to improve real-time treatment monitoring and risk stratification strategy to offer clinicians vital timely information to tailor treatment regimens for individual NPC patients. We expect the NGS mutational profiling capability to enhance our future understanding of the genetic basis for metastatic disease and identify predictive molecular signatures associated with drug resistance and poor treatment outcomes.

## Supplementary information

Supplementary document

## Data Availability

The data used in this paper is available from the corresponding author upon reasonable request.
