# Supplementary material for: Clinical utility of serial analysis of circulating tumour cells for detection of minimal residual disease of metastatic nasopharyngeal carcinoma
Source: Br J Cancer. 2020 May 6;123(1):114–25. doi: 10.1038/s41416-020-0871-1 (PMC7341819; doi:10.1038/s41416-020-0871-1)
Supplement: Supplementary file 1 — Supplementary document [file 41416_2020_871_MOESM1_ESM.docx]

**Figure S1: Serial timeline for blood sampling**


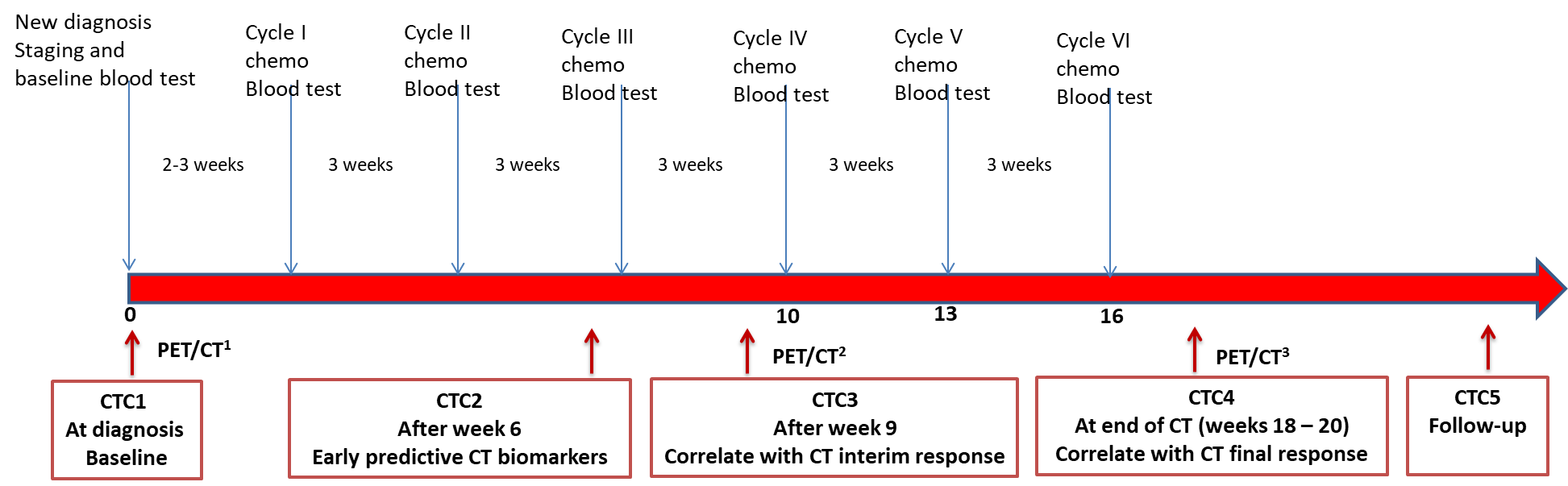


**
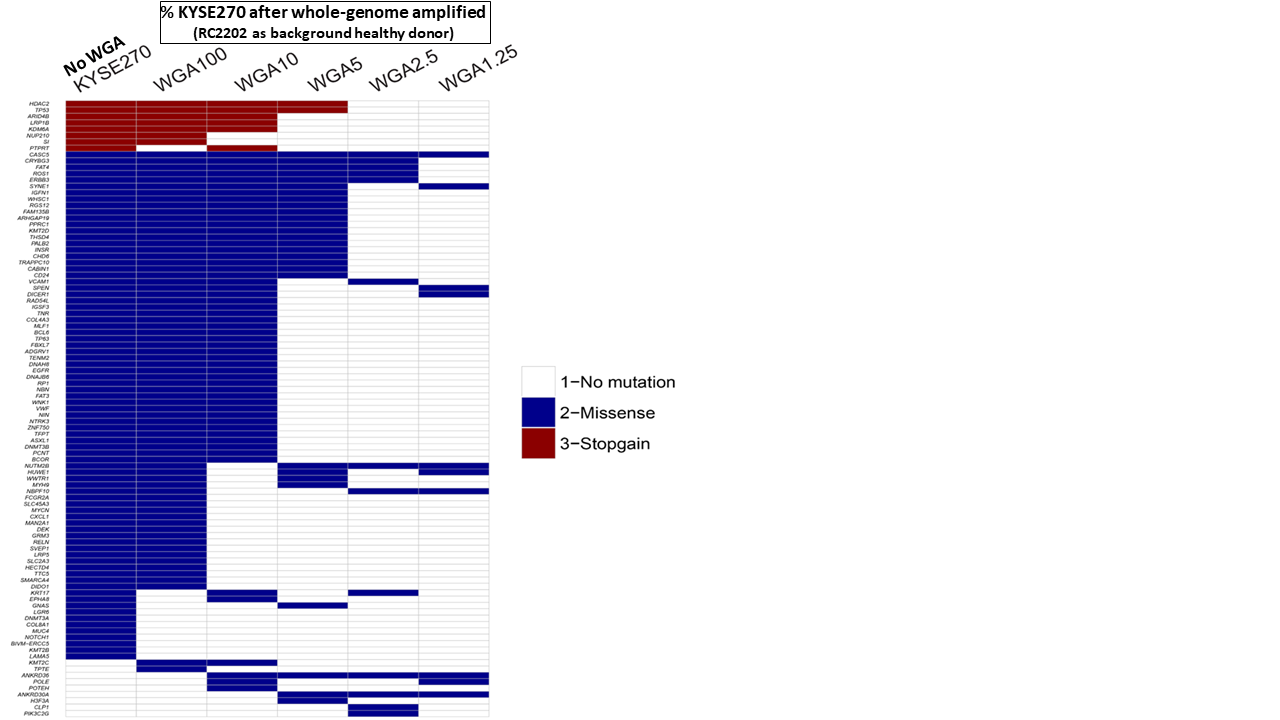
**

**Figure S2: Landscape of varying percentages of KYSE270 after WGA compared to KYSE270 without WGA in spike-in proof-of-concept experiment.**

**Table S1: Summary of baseline clinical information in 21 NPC patients.**

| **Parameters** | **N (%)** |
| --- | --- |
| **Age (median and range)** | **57 (13-70)** |
|  |  |
| **Gender** |  |
| Male | 17 (81) |
| Female | 4 (19) |
|  |  |
| **T classification** |  |
| T1 | 3 (14.3) |
| T2 | 1 (4.8) |
| T3 | 12 (57.1) |
| T4 | 5 (23.8) |
|  |  |
| **N classification** |  |
| N0 | 1 (4.8) |
| N1 | 5 (23.8) |
| N2 | 6 (28.6) |
| N3 | 9 (42.9) |
|  |  |
| **M classification** |  |
| M0 | 9 (42.9) |
| M1 | 12 (57.1) |
|  |  |
| **Extent of metastatic disease** |  |
| Liver | 10 (47.6) |
| Lung | 8 (38.1) |
| Distant lymph nodes | 10 (47.6) |
| Bone | 12 (57.1) |
| Other | 1 (4.8) |

According to the 7th edition of the AJCC/UICC staging system; WHO = World Health Organization; NPC = nasopharyngeal carcinoma.

| **Table S2: Targeted sequencing data statistics** | | | | | | |  |  |  |
| --- | --- | --- | --- | --- | --- | --- | --- | --- | --- |
|  | | | | | | |  |  |  |
| **Sample** | **Total reads** | **% Unique reads** | **% Off-bait reads** | **Mean**  **on-target coverage** | **% Usable bases on target** | **% Target bases 10X** | **% Target bases 30X** | **% Target bases 50X** | **% Target bases 100X** |
| No WGA KYSE270 | 13384990 | 0.77 | 0.37 | 102.67 | 0.38 | 0.95 | 0.85 | 0.72 | 0.41 |
| No WGA RC2202 | 12571432 | 0.77 | 0.37 | 97.03 | 0.38 | 0.95 | 0.86 | 0.73 | 0.42 |
| WGA -00%  RC2202 | 11960947 | 0.76 | 0.43 | 83.06 | 0.34 | 0.84 | 0.69 | 0.57 | 0.32 |
| WGA-100%  KYSE270 | 12181095 | 0.77 | 0.38 | 90.10 | 0.36 | 0.84 | 0.69 | 0.57 | 0.33 |
| WGA-10% KYSE270 | 146283995 | 0.69 | 0.37 | 982.60 | 0.33 | 0.96 | 0.93 | 0.91 | 0.86 |
| WGA-5% KYSE270 | 167906130 | 0.67 | 0.36 | 1097.00 | 0.33 | 0.96 | 0.94 | 0.92 | 0.88 |
| WGA-2.5% KYSE270 | 128887133 | 0.69 | 0.36 | 867.46 | 0.33 | 0.96 | 0.93 | 0.90 | 0.85 |
| WGA-1.25% KYSE270 | 124785290 | 0.68 | 0.35 | 833.98 | 0.33 | 0.96 | 0.93 | 0.90 | 0.85 |
